# Diabetes, prostate cancer screening and risk of low- and high-grade prostate cancer: an 11 year historical population follow-up study of more than 1 million men

**DOI:** 10.1007/s00125-016-3972-x

**Published:** 2016-05-17

**Authors:** Rachel Dankner, Paolo Boffetta, Lital Keinan-Boker, Ran D. Balicer, Alla Berlin, Liraz Olmer, Havi Murad, Barbara Silverman, Moshe Hoshen, Laurence S. Freedman

**Affiliations:** Unit for Cardiovascular Epidemiology, The Gertner Institute for Epidemiology and Health Policy Research, Sheba Medical Center, Tel Hashomer, 52621 Israel; Sackler Faculty of Medicine, School of Public Health, Department of Epidemiology and Preventive Medicine, Tel Aviv University, Tel Aviv, Israel; Patient Oriented Research, The Feinstein Institute for Medical Research, Manhasset, NY USA; Tisch Cancer Institute, Icahn School of Medicine at Mount Sinai, New York, NY USA; Israel Center for Disease Control (ICDC), Israel Ministry of Health, Tel Hashomer, Israel; School of Public Health, Faculty of Social Welfare and Health Sciences, Haifa University, Haifa, Israel; Clalit Research Institute, Clalit Health Services, Tel Aviv, Israel; Public Health Department, Ben-Gurion University of the Negev, Beer Sheva, Israel; Biostatistics Unit, Gertner Institute for Epidemiology and Health Policy Research, Sheba Medical Center, Tel Hashomer, Israel

**Keywords:** Diabetes, Ethnic origin, Gleason score, Prostate cancer, PSA screening, PSA testing, Socioeconomic status

## Abstract

**Aims/hypothesis:**

An inverse association has consistently been shown between diabetes and prostate cancer incidence. We investigated whether lower prostate cancer incidence among men with diabetes is attributable to lower detection due to prostate cancer screening patterns.

**Methods:**

We studied a population-based historical cohort of 1,034,074 Israeli men aged 21–90 years, without a previous history of cancer. The cohort was followed-up from 2002 to 2012, according to diabetes morbidity, for frequency of prostate-specific antigen (PSA) testing, mean PSA values and detection of prostate cancer, after adjustment for age, ethnic origin, socioeconomic status and PSA testing.

**Results:**

In January 2002, 74,756 men had prevalent diabetes. During the 11 year follow-up, 765,483 (74%) remained diabetes-free and 193,835 developed diabetes. Approximately 10% more PSA screening was performed in men with than without diabetes, but the rate of PSA positivity (>4 μg/l) was 20% lower in men with diabetes. PSA values were already significantly lower in men who developed diabetes than in those who did not, 3 years before diabetes diagnosis. Reduced prostate cancer risk was observed among men with incident diabetes only for low–moderate grade tumours (Gleason score 2–6: adjusted HR 0.83; 95% CI 0.77, 0.89). No association was observed for high-grade tumours (Gleason score 7–10: HR 0.99; 95% CI 0.88, 1.11).

**Conclusions/interpretation:**

Our findings suggest that diabetes comorbidity is a factor to be considered in prostate cancer screening strategies, and specifically in the interpretation of PSA levels. Furthermore, our demonstration of reduced incidence of low–moderate grade but not high-grade prostate cancer tumours among men with diabetes supports the possibility that low PSA levels, rather than lower tumour risk, explains the observed reduced incidence of prostate cancer in men with diabetes.

***Trial registration*:**

ClinicalTrials.gov NCT02072902

## Introduction

Many studies have shown an inverse association between diabetes and prostate cancer incidence; recent publications include two large meta-analyses of case–control and cohort studies [1], the European Prospective Investigation into Cancer and Nutrition (EPIC) study [2], and nationwide studies from Sweden [3] and Australia [4]. Similar data have been reported in Israel [5–7]. A number of US investigations have reported that the strength of this negative association increases with increased duration of diabetes [8–10], while others have shown it to remain constant with diabetes duration [11]. By contrast, studies in Chinese populations have shown positive associations between diabetes and prostate cancer [12].

Only a few investigations of the association between diabetes and prostate cancer have stratified by grade of prostate cancer. The EPIC study did not find evidence that the inverse association differed by disease grade [2]; however, grade was available for fewer than half of the cases. Two Japanese studies [13, 14], a Korean study [15], a Swedish study [16] and a US retrospective study [17] reported positive associations between diabetes and aggressive prostate cancer. A prospective US study [18] with 9 years of follow-up showed that diabetes was inversely associated with early stage prostate cancer, but not associated with aggressive prostate cancer. A systematic review identified four studies that analysed the association between diabetes and prostate cancer according to grade. A meta-analysis of these studies revealed an inverse association for both low-grade and high-grade cancer, which was stronger for the former [19].

Studies conducted in the USA have reported increased screening rates for prostate cancer following diabetes diagnosis [20]. Cross-sectional studies conducted in Japan [21] and Germany [22] showed lower prostate-specific antigen (PSA) levels in men with diabetes than in men without the disease. In addition, a US prospective cohort study of randomly selected men reported less annual changes in PSA in men with than without diabetes [23]. Longer diabetes duration [24] and more severe diabetes [22] were found to be associated with lower PSA levels. However, none of the above studies investigated whether diabetes-specific patterns of surveillance or of PSA levels may contribute to an inverse association between diabetes and prostate cancer incidence.

We investigated whether the observed lower incidence of prostate cancer among men with diabetes may be a consequence of prostate cancer screening. To this end, we estimated HRs for prostate cancer among men with diabetes, according to grade of prostate cancer, and compared rates of prostate cancer screening and PSA level distributions among men with and without diabetes.

## Methods

The study is based on electronic records from the largest health maintenance organisation in Israel, Clalit Health Services, which insures and provides healthcare to 53% (4.3 million) of the nation’s population. All men aged 21 to 89 years on 1 January 2002 (the date of study entry), without a previous history of cancer, were included in a closed historical cohort that was followed until 31 December 2012 for the incidence of diabetes. All men were followed-up for prostate cancer incidence, which was ascertained by record linkage to the Israel National Cancer Registry (INCR). The INCR maintains records of all solid malignancies diagnosed since 1960, and is updated annually according to mortality records. Reporting to the registry has been mandated by law since 1982, and the data come from multiple sources: pathological reports, hospital discharge medical files, oncology institutes, and outpatient and private clinics. Completeness of reporting reaches 95% for solid tumours [25].

### Definitions of diabetes

We separately classified prevalent and incident diabetes. Incident diabetes was defined as fulfilment of at least one of the following six criteria after January 1, 2002: (1) case of diabetes included in the Clalit Chronic Disease Registry; (2) a physician’s diagnosis of diabetes, together with one plasma glucose test >6.9 mmol/l within 12 months; (3) two plasma glucose measurements >6.9 mmol/l within 12 months; (4) one measurement of HbA_1c_ ≥6.5%; (5) 2 h plasma glucose level during an oral glucose tolerance test ≥11.1 mmol/l; and (6) three or more purchases of glucose-lowering medication within 12 months. The date of the earliest defining criterion was considered the date of diabetes diagnosis. The prevalent diabetes group comprised patients who were recorded in the Clalit Chronic Disease Registry as having diabetes on 1 January 2002 or who fulfilled criterion no. 6 above (information on medication use was available from 1998) before 1 January 2002.

The classification of diabetes used in the current study is similar to that used by Clalit Health Services in a study of diabetes incidence and prevalence that reported high internal validity of the definition of diabetes [26].

### Grading of prostate cancer

Grading of prostate cancer was according to the method defined by the Middle East Cancer Consortium [27]. Well differentiated prostate cancers (i.e. Gleason scores 2–4; Gleason patterns 1,2; histological grade I) were coded 1; moderately differentiated prostate cancers (i.e. Gleason scores 5,6; Gleason pattern 3; histological grade II) were coded 2; and poorly differentiated prostate cancers (i.e. Gleason scores 7–10; Gleason patterns 4,5; histological grade III) were coded 3.

### Calculating HRs for prostate cancer among men with diabetes

Preliminary analyses were performed to examine distributions of the main variables, check the plausibility of the values reported and determine categories to be used in the analysis. To estimate HRs for prostate cancer between those with and without diabetes, Cox regression models were applied, with adjustments for age (in 5 year age groups), ethnic origin (country of birth or mother’s country of birth: Ashkenazi Jews [those born in Russia, Eastern Europe, Europe, America or South Africa]; Sephardic Jews [those born in mid and northern Africa or the Middle East]; Yemenite Jews; Ethiopian and Central African Jews; Israeli Jews [including also Israeli-born Jews whose mothers’ birthplaces were unknown]; and Israeli Arabs) and socioeconomic status (SES) according to affiliation to local Clalit Health Services clinics (low, medium, high). Missing information on SES was categorised as ‘missing’.

The time origin for the model was 1 January 2002. The HR for individuals with prevalent diabetes (diagnosed before 2002) was estimated separately from that of those with incident diabetes. For incident diabetes, diabetes status (yes/no) was included in the model as a time-dependent covariate. This approach avoids the problem of immortal time bias that has affected other research investigations of the relationship between diabetes and cancer [28]. To account for potential reverse causation, further time-dependent variables were entered to distinguish between the first year, the second year and the subsequent years following diabetes diagnosis. Men who were not diagnosed with prostate cancer were censored at the earliest of the following events: date of death, 90th birthday or 31 December 2012. When estimating the HR for prostate cancer of a certain grade, all cases of prostate cancer with a different grade were censored at the time of diagnosis.

An additional analysis was performed that also adjusted for PSA screening. For this analysis, only follow-up years 2005–2012 were considered, with adjustment for PSA screening during 2002–2003. The rationale was that if PSA was tested in 2002–2003, at least 2 years before prostate cancer ascertainment, then the test was evidently indicated for screening and not for diagnostic purposes. Only men without a diagnosis of prostate cancer as of 1 January 2005 were included in this analysis. Men who reached the age of 90 years or died before 1 January 2005 were excluded.

The analyses were performed using PROC PHREG of SAS version 9.3 for PC (SAS Institute, Cary, NC, USA). We overcame the problem of excessively long run-times that could occur in the application of this model to a massive dataset, by grouping events into 6 month periods and using the Breslow method for handling tied event times [29]. This approach is similar to using an exponential piecewise model with periods of 6 months.

### Assessment of prostate cancer screening according to diabetes duration

To investigate whether men with diabetes in our study population underwent increased surveillance for prostate cancer, we assessed the proportions of men aged 50 years and older who underwent PSA testing during a given year (2008) according to the year of diabetes diagnosis (2003, 2007, 2008 and 2009) as well as of men who remained diabetes-free during the study period 2002–2012. The years of diabetes diagnosis were selected to depict PSA screening in the period just before and just after diagnosis (2007, 2009), as well as several years after (2003).

### Comparison of PSA values between men with and without diabetes

To compare mean PSA values between men with and without diabetes, before and after diagnosis, we examined for each year during 2002–2012, mean PSA values of men diagnosed with diabetes in 2008 and of those not diagnosed with diabetes by the end of 2008. To make the same comparison over a longer period following the diagnosis of diabetes, we examined mean PSA values during 2002–2012 in men diagnosed with diabetes before 2002 (prevalent diabetes group), men diagnosed in 2002 and men not diagnosed by the end of 2012. Differences between groups were tested statistically using mixed linear models applied to log-transformed PSA values, adjusted for age.

Our investigations have been approved by the responsible ethics committee (institutional review board) and were carried out in accordance with the Declaration of Helsinki as revised in 2008.

## Results

Among the 1,034,074 men included, 765,483 (74%) remained diabetes-free during the 11-year follow-up, 74,756 had prevalent diabetes and 193,835 incident diabetes (Table 1). A total of 14,099 prostate cancer events were registered in INCR during that time. Among men diagnosed with prostate cancer, the proportion of high Gleason scores (7–10) was greater for those with than without diabetes, both for those with follow-up in 2002–2012 and the subgroup followed in 2005–2012 (Table 2). After adjusting for age, SES and ethnic origin, the HR for all prostate cancer was 1.65 for men with incident diabetes (compared with men without diabetes) during the first year following diabetes diagnosis (95% CI 1.55, 1.76); however, the HR decreased and was significantly lower than 1 by the third and subsequent years following diabetes diagnosis (HR 0.89, 95% CI 0.84, 0.95), as it was for the prevalent diabetes group (HR 0.80, 95% CI 0.76, 0.85; Table 3). An HR significantly lower than 1 during the third and subsequent years following diagnosis was also observed for low-medium grade prostate cancer (Gleason score 2–6; HR 0.83, 95% CI 0.77, 0.89); however, for high-grade cancer (Gleason score 7–10), the estimated risk from the third year on after diagnosis was hardly different from that of men without diabetes, with HR 0.99 (95% CI 0.88, 1.11; Table 3). The results of the sub-analysis that controlled for PSA testing during 2002–2003 were similar to those for the full period analysis (Table 3).

**Table 1:** Characteristics of the study population of men aged 21–89 years, according to diabetes status

Characteristics	Diabetes-free *n* = 765,483	Prevalent diabetes *n* = 74,756	Incident diabetes *n* = 193,835
Age (years), mean ± SD	41.6 ± 15.9	62.6 ± 12.8	56.1 ± 14.5
Ethnic origin^a^ (%)			
Ashkenazi Jews	27.05	33.13	31.74
Sephardic Jews	25.47	29.38	27.94
Israeli Jews	24.45	16.80	18.23
Israeli Arabs	19.12	14.95	16.70
Yemenite Jews	2.54	4.93	4.01
Ethiopian and Central African Jews	1.37	0.81	1.38
SES (%)			
Low	40.57	40.85	42.31
Medium	38.24	39.82	37.91
High	17.43	16.54	17.00
Missing	3.77	2.78	2.78
Smoking (%)			
Never smoker + missing	57.73	58.87	49.48
Past + current smoker	42.27	41.13	50.52

^a^According to country of birth or mother’s country of birth

**Table 2:** Prostate-cancer-related characteristics according to glycaemic group

Characteristics	Follow-up: 2002–2012 *n* = 1,034,074			Follow-up: 2005–2012 *n* = 997,440		
	Diabetes-free *n* = 765,483	Prevalent diabetes *n* = 74,756	Incident diabetes *n* = 193,835	Diabetes-free *n* = 746,551	Prevalent diabetes *n* = 65,001	Incident diabetes *n* = 185,888
Person-years	9,002,033	640,388	982,263	6,268,686	431,200	876,460
Prostate cancer frequency, *n* (crude rate per annum × 10^3^)	9,714 (1.08)	1,672 (2.61)	2,713 (2.76)	6,939 (1.11)	1,113 (2.58)	2,283 (2.60)
Gleason score for prostate cancer; *n* (%)						
2, 3, 4 (1^a^)	177 (1.8)	33 (2.0)	31 (1.1)	81 (1.2)	14 (1.3)	15 (0.7)
5, 6 (2^a^)	5,983 (61.6)	852 (51.0)	1,567 (57.8)	4,666 (67.2)	623 (56.0)	1,375 (60.2)
7–10 (3^a^)	1,742 (17.9)	358 (21.4)	623 (23.0)	1,319 (19.0)	269 (24.2)	539 (23.6)
Unknown	1,812 (18.7)	429 (25.6)	492 (18.1)	873 (12.6)	207 (18.6)	354 (15.5)
PSA screening in 2002–2003						
No PSA screening				677,412 (90.7)	47,025 (72.4)	144,003 (77.5)
PSA screen in 1 out of 2 years				50,505 (6.8)	12,990 (20.0)	29,671 (16.0)
PSA screen in 2 out of 2 years				18,634 (2.5)	4,986 (7.7)	12,214 (6.6)

^a^1, 2, 3: According to Middle East Cancer Consortium manual of coding and staging [27]

**Table 3:** Number of prostate cancers (NC), HRs and 95% CIs for HR for prostate cancer incidence in diabetic compared with non-diabetic men according to 1st, 2nd and the rest of the follow-up years from diabetes diagnosis

Gleason score category	Follow-up: 2002–2012^a^ HR (95% CI) 14,099 prostate cancer events including those in non-diabetics					Follow-up: 2005–2012^a^ PSA-adjusted^b^ HR (95% CI) 10,335 prostate cancer events including those in non-diabetics				
	Incident diabetes *n*=193,835				Prevalent diabetes *n*=74,756	Incident diabetes *n*=185,888				Prevalent diabetes *n*=65,001
	1st year	2nd year	3rd year+	First 10 years		1st year	2nd year	3rd year+	First 7 years	
All:										
NC	767	354	1, 592		1, 672	460	276	1, 547		1, 113
HR	1.65	0.93	0.89	0.97	0.80	1.48	0.94	0.89	0.98	0.77
(95% CI)	(1.55, 1.76)	(0.84, 1.03)	(0.84, 0.95)	(0.93, 1.02)	(0.76, 0.85)	(1.37, 1.61)	(0.83, 1.06)	(0.84, 0.94)	(0.94, 1.02)	(0.72, 0.82)
2–6:										
NC	420	217	961		885	271	179	940		637
HR	1.47	0.89	0.83	0.90	0.71	1.32	0.90	0.82	0.91	0.68
(95% CI)	(1.35, 1.60)	(0.77, 1.01)	(0.77, 0.89)	(0.85, 0.95)	(0.66, 0.76)	(1.19, 1.47)	(0.78, 1.05)	(0.77, 0.89)	(0.85, 0.96)	(0.62, 0.74)
7–10:										
NC	180	66	377		358	119	51	369		269
HR	2.09	0.86	0.99	1.08	0.92	2.05	0.88	1.00	1.13	0.93
(95% CI)	(1.83, 2.38)	(0.66, 1.10)	(0.88, 1.11)	(0.98, 1.20)	(0.82, 1.03)	(1.75, 2.39)	(0.67, 1.17)	(0.89, 1.13)	(1.02, 1.25)	(0.82, 1.06)
Unknown:										
NC	167	71	254		429	70	46	238		207
HR	1.78	1.18	1.09	1.17	0.98	1.51	1.21	1.08	1.16	0.96
(95% CI)	(1.56, 2.03)	(0.94, 1.48)	(0.94, 1.26)	(1.03, 1.31)	(0.88, 1.09)	(1.23, 1.86)	(0.92, 1.61)	(0.93, 1.25)	(1.01, 1.30)	(0.82, 1.12)

^a^Adjusted for age category (5 year increments), SES and ethnic origin

^b^Adjusted for PSA screening during 2002–2003

NC, number of cases

**Fig. 1:**
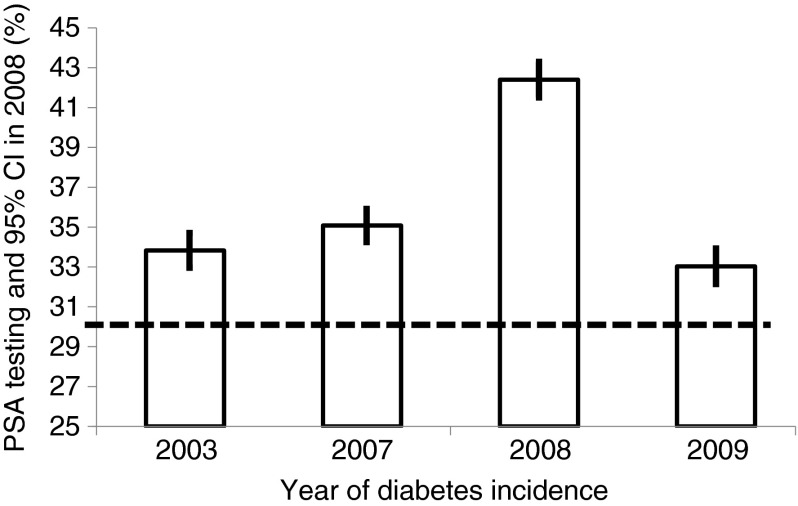
Percentage of PSA testing and 95% CI in 2008 in men with diabetes aged ≥50 years, according to year of diabetes incidence, adjusted for age, SES and ethnic group. The dashed line shows the 30.6% figure for PSA screening in 2008 for men not diagnosed with diabetes during the study period

Among men with incident diabetes aged 50 years and older, PSA screening percentages in 2008 were 33.8% for those diagnosed 5 years earlier (2003), 35.1% for those diagnosed in the previous year (2007), 42.4% for those diagnosed with diabetes in the same year (2008) and 33.0% for those diagnosed the year after (2009). By contrast, for men not diagnosed with diabetes during the study period, 30.6% were screened for PSA in 2008 (Fig. 1). Thus, among men diagnosed with diabetes several years earlier, the proportion screened in 2008 (33.8%) was 10% higher than among those who were not diagnosed with diabetes during the study (30.6%). Adjusting for age, SES and ethnic origin in a regression model did not change this finding (not shown).

**Fig. 2:**
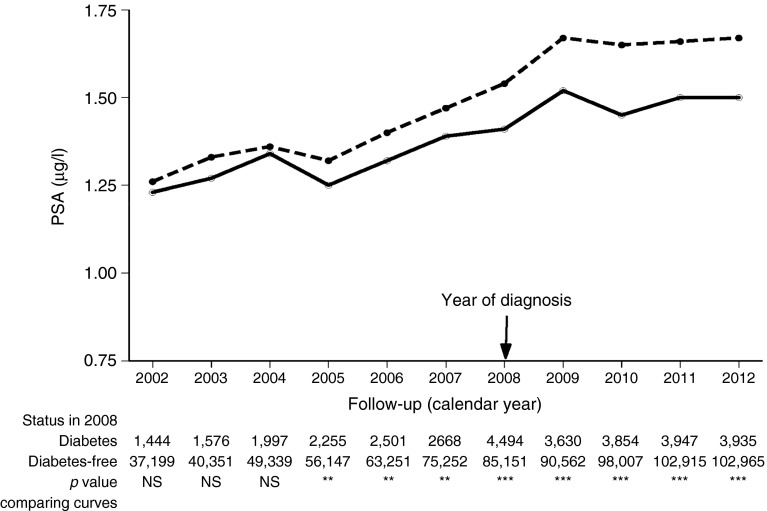
Geometric mean PSA levels by calendar year for men aged 50–70 years in 2008, who were diagnosed with diabetes in the year 2008 (solid line) and men free of diabetes at the end of 2008 (dashed line), adjusted to data for a 60-year-old man. The number of observations in each year is shown below the graph; NS, not significant; *p* > 0.05; **p < 0.01; ***p < 0.001

**Fig. 3:**
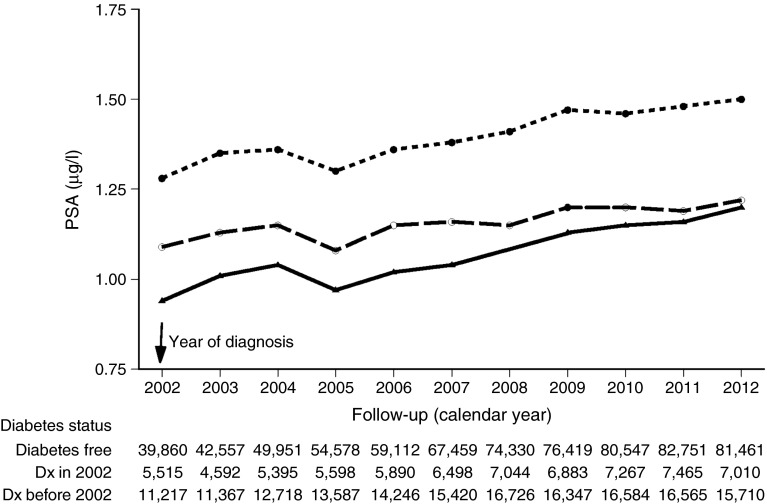
Geometric mean PSA levels by calendar year for men aged 50–70 years in 2002 who were tested for PSA, according to diabetes status in 2002, adjusted to data for a 60-year-old man. Solid line, diabetic men diagnosed before 2002, dashed line, diabetic men diagnosed in 2002; dotted line, men not diagnosed with diabetes during 2002–2012. The number of observations in each year is shown below the graph. The curves of the diabetic groups are significantly different from the curve of the non-diabetic group at each time point (*p* < 0.001). Dx, diagnosis

Mean PSA values were significantly lower for men with diabetes, even prior to diabetes diagnosis (e.g. 2005 in Fig. 2). Moreover, although mean values increased over time as individuals aged, they increased more rapidly among men without diabetes than among men with diabetes (Figs 2, 3). For men diagnosed with diabetes 10 years previously, mean PSA values were similar to those of men with long-term diabetes (the prevalent diabetes group; Fig. 3), and for both were approximately 20% lower than in men who were diabetes-free. Over the period 2002–2012, the proportion of men with positive PSA values (above 4 μg/l) was 16–18% among those diagnosed with diabetes in 2002, 13–18% among those with prevalent diabetes and 17–23% in men free of diabetes. Among men with incident diabetes, compared with men who remained diabetes-free, the proportion with positive PSA tests was 8% lower (ratio 0.92) in the year they were diagnosed (2002) and decreased to about 20% lower (ratio ∼0.80) at about 6–8 years after diagnosis, thus reaching the same ratio as for men with prevalent diabetes (Table 4).

**Table 4:** Numbers and percentages of screened men aged 50–70 years with PSA test values >4 μg/l according to year of test and diabetes status

Year	Diabetes-free during follow-up		Diabetes diagnosed in 2002		Prevalent diabetes: diagnosed before 2002	
	*n* ^a^	%PSA >4 μg/l	*n* ^a^	%PSA >4 μg/l^b^	*n* ^a^	%PSA >4 μg/l^b^
2002	39,860	17.2	5,515	15.9 (0.92)	11,217	12.6 (0.73)
2003	42,357	18.5	4,592	16.7 (0.90)	11,367	15.0 (0.81)
2004	49,951	18.3	5,395	16.8 (0.92)	12,718	15.2 (0.83)
2005	54,578	17.4	5,598	15.4 (0.89)	13,587	14.0 (0.80)
2006	59,112	19.1	5,890	16.9 (0.88)	14,246	15.7 (0.82)
2007	67,459	19.2	6,498	17.3 (0.90)	15,420	15.1 (0.79)
2008	74,330	19.8	7,044	15.9 (0.80)	16,726	16.1 (0.81)
2009	76,419	21.2	6,883	17.4 (0.82)	16,347	17.7 (0.83)
2010	80,547	21.2	7,267	18.3 (0.86)	16,584	17.0 (0.80)
2011	82,751	21.9	7,465	17.6 (0.80)	16,565	17.2 (0.79)
2012	81,461	22.6	7,010	18.3 (0.81)	16,710	17.6 (0.78)

^a^Numbers with a PSA test

^b^In parentheses, ratio of %PSA >4 μg/l to that in men without diabetes

## Discussion

The main findings of this study were as follows: (1) in the third and subsequent years following diabetes diagnosis, the HR for prostate cancer was significantly less than 1 for low–moderate grade prostate cancer (HR 0.83, 95% CI 0.77, 0.89) but not for high-grade prostate cancer (HR 0.99, 95% CI 0.88, 1.11); (2) an approximately 10% higher PSA screening rate was found among men with diabetes; (3) men who developed diabetes had lower PSA levels prior to a diagnosis of diabetes, with the gap widening during the first 10 years following diagnosis; and (4) an approximately 20% lower rate of PSA positivity (>4 μg/l) was found in men with diabetes from 6–8 years after diagnosis onwards. Other factors being equal, the increased rate of screening among men with diabetes would be expected to result in an HR >1 for screening-detected cancers. However, together with the lower PSA positivity, an HR <1 would be expected. In fact, one would expect an overall HR for prostate cancer of approximately 0.8 × 1.1 = 0.88 among men with diabetes, similar to the observed results.

Lower androgen levels have been suggested as an explanation for lower PSA levels in men with diabetes [30]. Testosterone has been shown to be positively associated with PSA levels [31], and men with type 2 diabetes are known to have lower testosterone levels [32].

The finding of lower PSA levels among men with diabetes supports later detection, as well as a lower probability of biopsy performance, as plausible explanations for lower incidence rates of prostate cancer in men with diabetes. These explanations are supported by our observation that the inverse association was found only for low–moderate grade tumours, and not for high-grade tumours. Assuming that the PSA-level threshold for referral for biopsy is the same for men with and without diabetes, then men with diabetes would presumably be less likely to be referred for biopsies than men without diabetes, for the same potential tumour severity. Among men with low-grade tumours, fewer men with diabetes would be expected to be referred for biopsy; yet, no such difference would be expected between men with and without diabetes for high-grade tumours. In the REDUCE trial, in which all participants underwent biopsies at 2 and 4 years after trial initiation, regardless of PSA concentrations, diabetes was not associated with prostate cancer risk [33]. Other studies that compared biopsies of men with and without diabetes reported higher grade tumours for the former group [34]. The implication is that different interpretations of PSA levels may be relevant to men with diabetes.

Similar to the findings and implications presented herein, a lower PSA concentration in obese men has been suggested as an explanation for delayed tumour detection, resulting in more aggressive disease at presentation [22, 35]. Furthermore, obesity was found to be a risk factor for prostate cancer at the time of biopsy, after adjusting for PSA levels and other clinical characteristics [36]. Lower PSA levels among overweight and obese men were shown to result in fewer biopsies [37]. Nevertheless, a sensitivity analysis on data from 2009 to 2012 that adjusted for BMI showed only modest, insubstantial changes in HRs of diabetes vs no diabetes for prostate cancer [7].

Differences in secondary prevention practices across geographical regions may explain some of the discrepancies observed among studies of diabetes and prostate cancer. In our population, the increased PSA surveillance among men with diabetes was dominated by the reduction in PSA positivity. However, the impact of PSA screening on the detection rate of prostate cancer among men with diabetes may differ according to regional and temporal trends in screening practice. High rates of prostate screening in the USA during the past 1–2 decades [37] may be related to the large number of US studies that reported an increased protective effect on prostate cancer with longer diabetes duration [7–9]. By contrast, diabetes duration was reported to be associated with an increased risk of prostate cancer in Taiwan [13]. Prostate screening in Taiwan was found to be more than twofold higher among men with than without diabetes. However, since the screening rate did not exceed 1%, a great effect of screening on incidence rates would not be expected. The rates of PSA screening in Israel, reported in this study, are similar to those in the USA [38]. Recently published data show that prostate cancer screening, as well as prostate cancer incidence, decreased following publication of the 2012 US Preventive Services Task Force Recommendations [39, 40]. Yet to be determined is whether this trend affects the association between diabetes and prostate cancer incidence, as changes may have occurred in the proportions of men with and without diabetes who are screened. Moreover, owing to the indolent nature of prostate cancer, it will take several years to verify the effect of current screening trends on prostate cancer mortality and on differences in rates between men with and without diabetes.

This was a population study, eliminating the problem of selection bias. Reverse causation was addressed by calculating HRs for prostate cancer among men with incident diabetes separately for the first and second years, and for the third year and after. The internal validity of this study was high, owing to the uniformity and standardised treatment in Israeli healthcare, especially in a single and the largest health maintenance organisation. There was no loss to follow-up and a relatively low frequency of missing data in the main studied variables.

In this analysis, we did not investigate the role of glycaemic control. Poor glycaemic control in men with type 1 diabetes was recently reported to be associated with lower PSA levels, independent of age, BMI, androgen levels, medication use and measures of diabetes severity [41]. In men with type 2 diabetes, poor glycaemic control has been found to be associated with a higher risk of prostate cancer detection [42] and with more aggressive prostate tumours [43]. Another limitation to the current study is that the proportion of prostate biopsies with undetermined Gleason grading reached 18–26%. In addition, we could not distinguish between non-smokers and men with missing smoking information in our database. We presume that such missing data would be more likely for non- or light-smokers. Nevertheless, a pooled analysis of 24 prospective cohort studies with over 21,579 prostate cancer cases found no association between smoking and prostate cancer incidence [44]. An effect of physical activity was not examined, as relevant data were not available in the database. No information on type of diabetes was available, but using age as a proxy for type 1 diabetes, the proportion of men younger than 35 years at diabetes incidence or at study entry was very low (3.1% and 2.3%, respectively) and no prostate cancer events occurred among these men.

Data on prostate cancer stage and fatality were lacking due to the absence of data on cause of death. Nonetheless, cause of death data is not always reliable [45]. Moreover, death following prostate cancer is dependent on age, comorbid conditions and length of follow-up. Since diabetes is one of the comorbid conditions to be considered, an assessment of death following prostate cancer diagnosis would be biased. Thus, high Gleason grade, as applied in this study, may be the most appropriate way to assess prostate cancer severity.

The relationship between diabetes, prostate cancer screening and incidence of prostate cancer is complex, and may differ across populations. Personalised screening strategies that are tailored to men’s individual risks and preferences have been advocated [46]. The findings of our current work suggest that diabetes comorbidity is a factor to be considered in prostate cancer screening strategies, and specifically in the interpretation of PSA levels. Furthermore, our demonstration of reduced incidence of low–moderate grade but not high-grade prostate cancer among men with diabetes supports the possibility that low PSA levels, rather than lower tumour risk, may explain the observed reduced incidence of prostate cancer. Prospective investigations are needed that assess PSA testing and concentrations, as well as biopsy performance and findings, in men with and without diabetes, in populations with different screening practices.

## Abbreviations

EPIC: European Prospective Investigation into Cancer and Nutrition
INCR: Israel National Cancer Registry
PSA: Prostate-specific antigen
SES: Socioeconomic status
